# Rapid Tranquillisation in Scunthorpe Acute Psychiatric Inpatient Service: A Re-Audit Highlighting Gender Differences, Clinical Trends, and NICE Compliance

**DOI:** 10.1192/bjo.2026.11742

**Published:** 2026-06-30

**Authors:** Homayun Shahpesandy, Abhimanyu Baruah, Katherine Freeman, Sharmin Bisma, Daniel Tuxworth

**Affiliations:** 1 Rotherham, Doncaster and South Humber NHS Foundation Trust, Scunthorpe, United Kingdom; 2 Lincolnshire Partnership NHS Foundation Trust, Lincoln, United Kingdom; 3 Rotherham, Doncaster and South Humber NHS, Scunthorpe, United Kingdom

## Abstract

**Aims:**

This re-audit evaluated local management of acutely disturbed and violent patients against NICE guideline NG10 (2015) and aimed to develop a targeted action plan. It compared trends between an initial audit in 2024 and a re-audit in 2025, focusing on gender differences in RT use, clinical presentation, and post-intervention care.

**Methods:**

A retrospective re-audit of 79 RT incidents between February 2024 and July 2025 was conducted on Mulberry Ward (Scunthorpe), an acute adult inpatient psychiatry ward and compared with 37 incidents from the 2024 audit (April 2022–July 2023). RT was defined per NICE NG10 (2015) as parenteral medication used for urgent sedation. Data were extracted from electronic medical records and Incident Report Form 1 (IR1). Analyses were performed using Microsoft Excel. Compliance with predefined standards of good clinical practice was assessed using a traffic-light system: green (≥80%), orange (60–79%), red (<60%). Gender-specific trends in demographics, RT frequency, clinical diagnoses, post-RT monitoring, and incident reporting were examined.

**Results:**

The 2024 audit included 17 patients (9 women, 8 men) and 37 RT incidents; the 2025 re-audit included 10 patients (7 women, 3 men) and 79 incidents. Female representation increased from 53% to 70%, with mean age decreasing from 43.8 to 30.3 years. RT frequency rose sharply among women (2.2 → 10.5 incidents per patient) and declined in men (2.0 → 1.5). Psychotic disorders remained common, but emotionally unstable personality disorder increased markedly (11.7% → 50%), predominantly affecting women. Secondary diagnoses, including poly-substance misuse (29.5% → 40%) and autistic spectrum disorder (23.5% → 40%), indicated rising clinical complexity.

Pharmacological management shifted substantially: lorazepam became the primary agent (43% → 69.6%), haloperidol-based regimens declined sharply, and aripiprazole injection increased (11% → 22.7%). Use of zuclopenthixol acetate and promethazine ceased in 2025. Self-harm (67%) and suicidal behaviour (13%) emerged as the main triggers for RT in women, while physical aggression declined to 16.5%. Seclusion use fell from 30% → 5%. NICE compliance remained excellent for risk assessment, patient rights, and documentation; however, post-RT monitoring, debriefing, medication review, and incident reporting remained below standard. Advance directives were absent in both audits.

**Conclusion:**

The re-audit demonstrates strong adherence to NICE standards and reduced reliance on seclusion, but persistent gaps in post-RT care and governance remain. The substantial increase in RT frequency among younger female patients with complex comorbidities highlights the need for gender-sensitive strategies, preventive interventions, trauma-informed care, and enhanced multidisciplinary follow-up. Findings support targeted service improvements and the ongoing role of audit in acute psychiatric care.

